# Avoidant Restrictive Food Intake Disorder in Pediatric Liver Transplant Patients

**DOI:** 10.1111/petr.70014

**Published:** 2024-12-27

**Authors:** Peyton Crest, Piper Stacey, Erin C. Accurso, Clara Deley, John Roberts

**Affiliations:** ^1^ Department of Surgery University of California San Francisco California USA; ^2^ Department of Psychiatry & Behavioral Sciences University of California San Francisco California USA; ^3^ Rhodes College Memphis Tennessee USA; ^4^ UMass Chan Medical School Worchester Massachusetts USA; ^5^ Philip R. Lee Institute for Health Policy Studies San Francisco California USA; ^6^ Clinical Nutrition University of California San Francisco California USA

**Keywords:** growth factors, growth patterns, pediatric, pediatric liver transplantation, pediatric transplantation, surgery

## Abstract

**Introduction:**

Following liver transplantation (LT), adequate nutrition is essential, as malnutrition may contribute to slower growth in pediatric patients and put patients at risk of complications following transplant. Avoidant Restrictive Food Intake Disorder (ARFID) is an eating disorder characterized by restrictive eating patterns that compromise nutrition. Patients with ARFID may have significant difficulty meeting nutritional needs due to fear of gastrointestinal distress, making it especially difficult to manage in patients following LT.

**Methods:**

We performed a retrospective chart review of de‐identified patients who received LT at our institution. Two patients with ARFID who had undergone LT were identified. Their diagnoses, clinical courses, and post LT outcomes are reported. A literature review of the presentation and diagnosis of ARFID in pediatric patients and nutritional management of pediatric LT patients was performed. No IRB review was required given the sample size of two patients, per UCSF IRB rules and regulations.

**Results:**

We present two unique cases of ARFID: one with onset prior to LT and one with onset following LT. Outpatient psychiatry treatment was essential for nutritional management for the patient who developed ARFID following LT. The other patient continues to see a dietitian given ongoing nausea that limits her oral intake but does not receive any psychiatric support.

**Conclusions:**

ARFID and selective eating patterns are rare but notable occurrences after pediatric LT, but they may also be underreported given the novelty of ARFID and the prevalence of gastrointestinal symptoms following transplant. Our case adds to the limited literature on ARFID in children following major surgical procedures and highlights the importance of interdisciplinary care and the importance of nutritional management in pediatric patients prior to and post LT.

**Abbreviations**
ARFID: avoidant restrictive food intake disorder
CBT: cognitive behavioral therapy
ED: emergency department
LT: liver transplantation
NG: nasogastric

## 1 Introduction

Liver transplantation (LT) is a well‐established, successful treatment for end‐stage liver disease in pediatric patients. Following transplantation, long term management of pediatric patients is essential and aims to ensure that pediatric patients follow normal growth patterns.

One particularly important aspect to pediatric growth and quality of life following LT is adequate nutrient intake [1]. Chronic liver diseases are characterized by impaired nutrient reabsorption and metabolism and increased proinflammatory cytokine levels, resulting in a hypermetabolic state [2]. As such, nearly 60% of pediatric patients undergoing LT assessment are malnourished [3]. Malnutrition prior to transplant sets children up for a need for “catch‐up” growth, which makes their recovery more challenging. Moukarzal et al. reported that prior to LT, 31% of children were below the fifth percentile for height [4].

After LT, the goal is to maintain adequate, age‐appropriate growth via oral feeding. However, in the setting of inadequate oral intake, enteral or parenteral nutrition may be initiated to meet nutrient needs [5]. Prioritization of protein and calorie intake is essential to avoid infection and muscle atrophy and promote wound healing. Food tolerance, however, may be limited in pediatric patients following LT, especially due gastroesophageal reflux with esophagitis, nausea, and early satiety [3]. Even when graft function is excellent, children often do not follow expected linear growth, and inadequate nutrition following transplant may contribute to slower growth [6, 7].

Avoidant Restrictive Food Intake Disorder (ARFID) is a relatively newer, heterogenous, eating disorder diagnosis in the fifth edition of the D*iagnostic and Statistical Manual of Mental Disorders* (*DSM‐5*) [8]. ARFID encompasses restrictive eating patterns that lead to compromised nutrition, significant stress, and interference with daily functioning in the absence of body image disturbance (Table 1). Notably, patients with ARFID may have difficulty meeting nutritional needs due to specific food fears or a fear of gastrointestinal distress or abdominal pain, which are often present following LT [9]. While abdominal pain has been reported to be a trigger for eating disturbances in ARFID patients, it is unclear whether these symptoms are due to surgical procedures [10, 11]. Moreover, ARFID spans several disciplines (e.g., mental health, nutrition, adolescent medicine, gastroenterology, social work, etc.), so it is more difficult to capture in studies focused on hepatic function and linear growth following transplant.

**TABLE 1:** DSM‐V diagnostic criteria for avoidant restrictive food intake disorder [7].

Avoidant restrictive food intake disorder diagnostic criteria
**1**. **An eating or feeding disturbance**, **which may include:**
Lack of interest in eating or food
Avoidance of food based on sensory characteristics
Concern about aversive consequences of food (e.g., choking and pain)
**2**. **One or more of the following:**
Significant weight loss (or failure to achieve expected weight gain or faltering growth in children)
Significant nutritional deficiency
Dependence on enteral feeding or oral supplements
Interference with psychosocial functioning
**3**. **Eating disturbances cannot be explained by:**
A current medical condition or another medical disorder
Lack of food or culturally sanctioned practices
A disturbance in the way one's body weight or shape is perceived

Here, we report two cases of avoidant restrictive eating habits and LT.

## 2 Case Report

### 2.1 Patient 1

An 11‐year‐old female underwent orthotopic LT for cirrhosis second to alpha‐1 antitrypsin (A1AT) deficiency. Four months following LT, she was diagnosed with ARFID.

**FIGURE 1:**
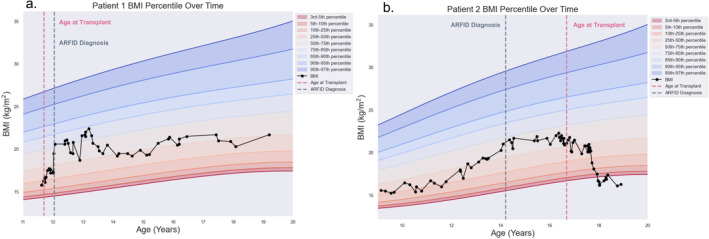
Patient 1 (a) and Patient 2 (b) BMI (kg/m^2^) over time. Percentile channels are based on Centers for Disease Control and Prevention Growth Charts for girls (2–20 years) BMI‐for‐age data [12].

Prior to LT, the patient was reported to be eating well with no difficulties with oral intake. Her body mass index was 15.77 kg/m^2^ (17th percentile), and she appeared to be well‐nourished (Figure 1a).

The LT was successful with no signs of graft rejection and normalization of liver function. She was readmitted on post‐operative day 10 to the emergency department (ED) for recurrent abdominal pain but otherwise had adequate oral intake. Her laboratory results were within normal limits, with the exception of slightly elevated creatinine (0.92 mg/dL). Upon admission, her oral intake decreased due to reported pain. To manage her abdominal pain and decreased oral intake, she was given IV boluses of Zofran (4 mg) and Dilaudid (0.6 mg), and a nasogastric (NG) feeding tube was placed, supplying 9.0 kcal/kg/day (1.0 g protein/kg/day) to supplement oral feedings, which was removed prior to discharge.

The patient's low appetite after transplant resulted in weight stagnation, with only a 100 g increase in weight 2 months following transplant, while 250 g per month would be expected in order to maintain historical trajectory [13]. She was reported to be far from reaching her caloric needs, taking only bites of food due to abdominal pain that was reported to “rise up” with each bite. Three months following ED discharge, she was readmitted due to dehydration, which was a result of repeated food refusal at home. She was diagnosed with hypokalemia and hypophosphatemia secondary to dehydration. Ultimately, the patient appeared to have a chronic cycle of dehydration, weakness, and fatigue, attributed to her abdominal pain and lack of adequate nutrition. She was supplemented with enteral feeding via an NG tube (40 kcal/kg/day, 1.5 g protein/kg/day), which she stated did not cause as much abdominal pain. Subsequently, the patient was diagnosed with ARFID by inpatient psychiatry services, given her weight loss, need for consistent enteral feeding, and interference with her psychosocial functioning. She also was diagnosed with generalized anxiety disorder, depression, and malnutrition. Studies ruled out alternative diagnoses, such as eosinophilic esophagitis and lymphoproliferative disorders.

Following her ARFID diagnosis, she had 4 subsequent readmissions to the ED within 6 months for bouts of nausea, intense abdominal pain, and difficulty eating. ED presentations were resolved with IV boluses of Compazine (2 mg) and Dilaudid (5 mg). Following her final discharge, she was reported to have adequate oral intake via liquid foods, which consisted of nutritional supplements. Despite multiple ED readmissions, the patient's weight rapidly increased multiple percentile channels (Figure 1a), likely due to rehydration.

**FIGURE 2:**
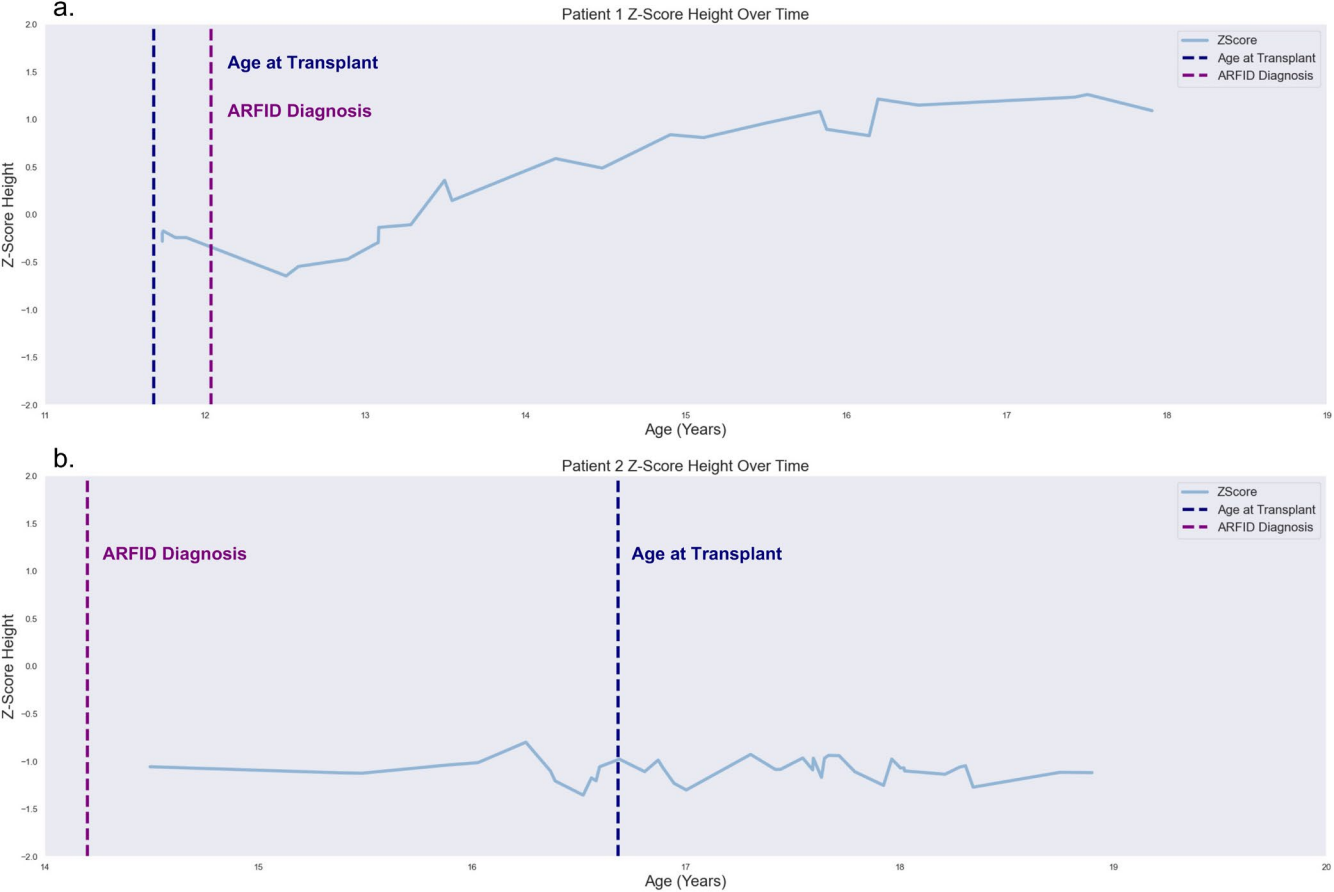
Patient 1 (a) and Patient 2 (b) linear growth height *Z*‐Scores over time. Linear interpolation was used for *Z*‐Score height, based on Centers for Disease Control and Prevention Stature‐for‐age data [12].

The patient and her family established care with a therapist for managing eating disturbances following her final ED discharge. Once her eating patterns normalized, her BMI normalized at the 25th percentile (Figure 1a), and her *Z*‐Score height increased from −0.5 to −0.1 (Figure 2a). She also was prescribed amitriptyline (50 mg/day) for chronic pain management. After 1 year of outpatient psychiatry treatment, she learned how to better control her pain symptoms through relaxation techniques and reported minimal pain flares.

Two years following transplant, she reported that her abdominal pain had resolved and presented no indications of malnutrition. At this time, her liver function tests and nutritional parameters are normal. Her graft function remains normal on a low dose immunosuppressive regimen of tacrolimus (target trough of 6–8 ng/mL) and has adequate nutritional intake. She has maintained a steady linear growth curve, with her height stabilizing at a *Z*‐Score of +1.0 (Figure 2a).

### 2.2 Patient 2

A 16‐year‐old female received LT for liver disease with portal hypertension secondary to multiple malignant liver masses. One month prior to transplant, the patient reported bouts of abdominal pain and decreased appetite.

Her BMI had been between the 15th and 25th percentiles until age 12, and she was reported to be a “very picky eater.” Approximately 2.5 years prior to LT, she was diagnosed with ARFID after a self‐referral to psychiatry. She received recommendations for sensory feeding and eating therapy sessions, which she inconsistently attended. During this time, she maintained her growth patterns, nearly reaching the 50th BMI percentile for her age. Other than selective eating patterns, she had normal linear growth and weight patterns (Figure 1b).

During LT, which was uncomplicated, an NG was placed and her tube feeds were advanced to meet 100% of her dietary needs. Pain, in conjunction with adequate oral nutrition, was the largest barrier to her discharge. However, within 10 days following LT, she tolerated her diet by mouth, and the NG tube was removed. The patient was discharged on a meal plan of 40 kcal/kg/day and 1.0 g protein/kg/day and her pain management team prescribed gabapentin (900 mg/day) to treat abdominal pain.

Two months following LT, she presented to the ED for recurrent nausea and decreased appetite due to intermittent, severe abdominal pain. She was evaluated by psychiatry consulting services and was reported to have distress/anxiety associated with pain. Her nutrient intake was found to be 25% or less of estimated energy/protein needs (40 kcal/kg/day, 1.0 g protein/kg/day), and she was diagnosed with malnutrition due to consistent weight loss. She was discharged with NG tube feedings to supplement her calorie intake and continued nightly feedings for 1 week following discharge. She was prescribed 16 mg/day Zofran for nausea.

At this time, the patient continues to report ongoing nausea that limits her oral intake, as she reports concern that eating highly caloric foods exacerbate gastrointestinal pain. Her oral intake and restrictive patterns have worsened (i.e., a greater number of foods are associated with abdominal pain) (Figure 1b), and her height has fluctuated around a *Z*‐Score of −1.0 (Figure 2b). Her graft function remains normal on a low dose immunosuppressive regimen of everolimus (target trough of 3–5 ng/mL). She currently sees a nutritionist and continues to take 16 mg/day Zofran but is not engaged in any outpatient psychiatric services despite provider recommendations.

## 3 Discussion

ARFID is a relatively new diagnosis, characterized by weight loss or inadequate growth, nutritional deficiencies, and a dependency on enteral feeding or oral supplements in the absence of body image disturbance. Because ARFID was not clearly defined and characterized until publication of the *DSM‐5* in 2013, discussion of its development and prognosis has been limited. As such, feeding aversion and ARFID symptoms following transplant are underreported. While poor growth trends and catch‐up growth have been reported in pediatric LT patients, psychological factors such as feeding aversion have not been considered [14]. Because feeding aversion spans several disciplines (e.g., mental health, nutrition, adolescent medicine, gastroenterology, social work, etc.), it is less likely to be captured by studies strictly focused on hepatic function and pediatric growth following transplant [15].

Moreover, pre‐transplant nutritional status, such as sarcopenia and dietary intake, has been extensively studied and associated with post‐transplant outcomes [16]. Indeed, aggressive nutritional support before LT improves graft function and patient survival. Preoperative weight and height *Z*‐Scores in pediatric LT patients predict catch‐up growth, and normal nutrition typically returns 6–12 months following LT [3]. Research on post‐transplant nutritional management and outcomes, however, is limited, especially for patients who do not return to normal nutrition patterns.

Here, we report two unique cases of pediatric LT patients. One patient developed ARFID following LT, and the second patient had a history of ARFID and selective eating patterns, which were exacerbated following LT. To our knowledge, these are the first cases to be reported on the co‐occurrence of selective eating behaviors and LT. The difference in patient ARFID prognosis, as illustrated by their BMI trends, is of note (Figure 1). Specifically, Patient 1 demonstrates a decrease in BMI following her ARFID diagnosis, but it eventually stabilized near the 50th percentile. This maintenance of her percentile rank also corresponds with establishing psychiatric care for her ARFID diagnosis. Patient 2, conversely, appeared to follow her BMI percentile channel until roughly 2 years following transplant, where her BMI dropped below the 3rd BMI percentile (*Z* = −1.96) (Figure 1b). Indeed, she continued to restrict her diet and did not establish psychiatric care for ARFID. Despite differences in BMI trends, both patients had good post‐operative hepatic function. Additionally, Patient 1 demonstrated a steady increase in her *Z*‐Score height following her ARFID diagnosis, while Patient 2 remained around a *Z*‐score of −1.0 (Figure 2). It is possible that differences in post‐transplant nutritional management (i.e., Patient 2 continues to maintain a restricted diet, while Patient 1 has an adequate nutritional intake) could impact these linear growth trends. While pre‐transplant linear height *Z*‐Score may be associated with linear growth impairment, attention to nutrition can impact post‐transplant growth [7]. Patient 2's poor linear growth trends emphasize the important of optimizing both pre‐transplant and post‐transplant nutrition.

One retrospective research study found that ARFID was associated with gastrointestinal symptoms (e.g., abdominal pain and constipation) [10]. An additional retrospective study of children diagnosed with ARFID found that 17.8% of patients reported abdominal pain as a trigger for their eating disturbance, although it was not clear whether or not the source of the reported abdominal pain was due to gastrointestinal disturbances or surgical procedures [11]. Likewise, the two patients presented were both diagnosed with chronic abdominal pain following LT, which was noted to be a significant source of their food restriction.

It is unclear whether LT influences the development of ARFID or serves as a risk factor. One hypothesis explores the loss of vagus innervation to the liver during LT. This isolation from autonomic regulatory control may influence appetite signaling, hunger, and eating behavior [17, 18]. Vagal afferent activity additionally impacts hepatic glucosensing, which has been suggested to be involved in the initiation of feeding behaviors rather than termination [19]. This presents an area of further research, especially given that ARFID arises from feeding initiation rather than termination, as would be expected for cues relating to satiety.

This case report highlights the need for awareness of aversive feeding and eating behaviors in children undergoing LT, whether these behaviors arise before or after transplant. Given that the nutritional behaviors of both patients in this report were mediated by abdominal pain, and LT has been associated with post‐traumatic stress symptoms that may increase physical pain, these cases demonstrate the importance of monitoring feeding and eating behaviors in pediatric patients after transplant [20]. Importantly, theoretical models indicate elevated anxiety as a predictor of avoidant eating behavior, and anxiety symptoms in children often increase following transplant [21]. This may further explain a potential link between ARFID and LT. Additional predisposing factors, such as food allergies and gastrointestinal disorders (e.g., irritable bowel syndrome [IBS]), may be especially pertinent for LT patients [22]. For children with a history of restrictive eating behaviors (e.g., picky eating during childhood), ARFID may be of greater risk following LT, given the temporary disruptions in nutrition. Further research should be conducted to determine if psychological disorders and eating behaviors during childhood serve as a risk factor for ARFID following LT.

Patients with ARFID are at severe risk of malnutrition, including electrolyte abnormalities [23]. Given that LT patients present increased caloric needs to avoid infection and promote wound healing, malnutrition exacerbated by ARFID poses additional concerns for patient outcomes following LT. Moreover, electrolyte abnormalities in pediatric patients following LT have been associated with risk of acute kidney injury, stressing the clinical implications of ARFID development post LT [24].

There are currently no randomized control trials that have investigated the efficacy of ARFID treatment among adolescents, and there is no clear standard treatment for ARFID [25]. Strategies to increase calorie intake in malnourished children often include tube feeding and intensive behavioral treatment interventions, such as exposure‐based cognitive‐behavioral therapy (CBT) [26]. LT patients may present additional complications due to increased nutritional needs, immunosuppression, and altered metabolism [3]. In this study, Patient 1 demonstrated improvement in eating behaviors following therapy and management of abdominal pain. Patient 2 was recommended to start occupational therapy for sensory integration concerns and feeding aversion.

Case 1 demonstrates the potential for developing food aversion following transplant, and Case 2 further highlights the need for clinicians to monitor feeding behaviors following transplant if patients present a history of food aversion. Given recent studies indicating that pediatricians and pediatric subspecialists are unfamiliar with ARFID, increasing awareness about this diagnosis may support improvements in medical care [27]. In particular, increased screening—especially in the context of medical conditions or procedures that increase risk for disordered eating or disruptions to eating—may help to identify individuals who would benefit from mental health or occupational services. Future research might examine the potential role of pre‐operative interventions to facilitate recovery for those at higher risk for post‐operative nutritional challenges (e.g., history of picky eating, low appetitive drive, high interoceptive sensitivity, and high anxiety).

This study presents a novel discussion on ARFID in pediatric LT patients. The study is limited by its access to only de‐identified data, which restricted the amount of information available for each patient's ARFID symptoms, diet, and treatment, as well as patient height prior to transplant. Following LT, it is important to monitor food aversion and feeding and eating behaviors in pediatric patients. Given the recent emphasis on Quality of Life (QOL) following LT, feeding aversion may be an emerging topic of interest [28]. As previous literature suggests, the success of LT depends on the collaboration of pediatricians, pediatric transplant hepatologists, transplant surgeons, dietitians, and mental health providers [29, 30]. The two cases presented exemplify the importance of such interdisciplinary care, especially between mental health providers and dietitians, and the importance of nutritional management in pediatric patients post LT.

## Conflicts of Interest

The authors declare no conflicts of interest.

## Data Availability

Data sharing is not applicable to this article, as no new data were created or analyzed in this study.
